# Aggression and locus of control in patients diagnosed with schizophrenia

**DOI:** 10.3389/fpsyt.2025.1600810

**Published:** 2025-07-21

**Authors:** Agnieszka Skowerska, Łukasz Czyżewski, Dorota Parnowska, Janusz Wyzgał, Andrzej Silczuk

**Affiliations:** ^1^ Department of Community Psychiatry, Medical University of Warsaw, Warsaw, Poland; ^2^ Department of Geriatric Nursing, Medical University of Warsaw, Warsaw, Poland; ^3^ Department of Psychiatry and Combat Stress, Military Institute of Medical Services, Warsaw, Poland; ^4^ Department of Nephrology Nursing, Medical University of Warsaw, Warsaw, Poland

**Keywords:** schizophrenia, aggression, control, locus of control, anxiety

## Abstract

**Purpose:**

Comparative analysis of data from patients diagnosed with schizophrenia and a control group from the healthy population regarding the level of aggression and anxiety, and the relationship between the locus of control, as well as analysis of the research hypothesis aimed at verifying whether higher levels of aggression occur in patients who place their sense of control outside themselves.

**Materials and methods:**

The research conducted in this study is a questionnaire-based, clinical-control study. It was carried out between 2019 and 2022 and included 61 patients with the ICD-10 diagnosis of schizophrenia who met the defined criteria and agreed to participate in the research project. The study group consisted of patients who were hospitalized in day psychiatric wards and 24-hour rehabilitation wards. Work material for the study group was collected using such tools as the ICD-10 International Classification of Diseases, GAF scale, BPAQ Aggression Questionnaire, STAI scale, questionnaire for measuring the locus of control, standardized Delta Questionnaire and a demographic survey.

**Results:**

The analysis revealed that the level of physical aggression was positively correlated with anxiety as a state (R=0.29; P<0.001), as a trait (R=0.32; P<0.001) and with an external sense of control (R=0.27; P<0.001). The level of verbal aggression was positively and weakly correlated with the sense of control (R=0.22; P<0.05) and weakly and negatively correlated with the lie scale (R=-0.24; P<0.001). Anger, hostility and general aggression were positively correlated with anxiety as a state (R=0.36; R=0.52; R=0.45; P<0.001, respectively) and as a trait (R = 0.50; R = 0.60; R = 0.56; P<00.001).

**Conclusions:**

The results indicate a statistically significant higher level of aggression in the group of patients diagnosed with schizophrenia compared to the control group representing the healthy population. The statistically significant differences in the patient group were observed in physical aggression, hostility, and the total aggression score. The results indicate that a higher level of aggression was observed in the subjects who placed the locus of control more externally.

## Introduction

The concept of locus of control (LOC) originates from Julian B. Rotter’s social learning theory (1954) (1). According to that concept, the sense of locus of control is one of the personality dimensions determining an individual’s autonomy and manifesting itself in various social behaviors. In his concept, Rotter distinguished between external and internal locus of control. A person takes action with the expectation that it will lead to a specific result, while the perceived relationship between behavior and the resulting outcome influences the level of expectation. According to Rotter’s concept, this means that people attribute the cause of events concerning themselves either to the results of their actions (internal locus of control) or to the effects of external events, being convinced that what happens to them is independent of the actions they take and remains beyond their control (external locus of control). Therefore, people with an internal locus of control develop constructive ways of dealing with adversities and obstacles in life. People with an externally located sense of control are often characterized by passivity and a lack of confidence in their abilities (2, 3) and a notable sensitivity to criticism, lack of confidence in themselves and their abilities. Their defense mechanisms are based on denial (4–6).

Multi-year studies of groups of schizophrenia patients demonstrate that people suffering from schizophrenia have a strong sense of external control, i.e. they lack a sense of control over their lives, their illness and various events, which they perceive as being caused by external factors. Research by Harrow and Ferrante (7) also indicates that external locus of control is more common when the illness is in the phase of intensification of psychopathological symptoms. Furthermore, individuals with an externally focused sense of control and a high level of anxiety may exhibit more severe aggressive behaviors due to perceiving the world as a threat (8–11).

People suffering from schizophrenia face many challenges in their everyday lives. Not only are they associated with a feeling of lack of control over their own life, but also with difficulties in functioning in the patients’ social life.

Retrospectively, clinical trials conducted so far in Poland and worldwide only assess the relationship between the sense of locus of control among patients with schizophrenia in a state of psychopathological exacerbation vs. patients with other psychiatric diagnoses (7, 12–15). The research conducted as part of this project is innovative. It also represents a search for a medical method that improves the quality of life of patients with schizophrenia and prevents relapses of the disease.

## Purpose

The research objective is to conduct a comparative analysis of results of the study on individuals diagnosed with schizophrenia and a control group representing the healthy population in terms of the level of aggression and anxiety and the relationship of the locus of control, as well as to evaluate and analyze the research hypothesis aimed at confirming or excluding the claim that higher levels of aggression will be present in patients who place their locus of control outside themselves. This study hypothesizes that patients with schizophrenia who exhibit an external locus of control will present higher levels of aggression.

## Materials and methods

The research conducted in this study is a questionnaire-based, clinical-control study. The study was approved by the Bioethics Committee at the Medical University of Warsaw (no. KB/160/2019). The research was conducted in accordance with the provisions of the Helsinki Declaration.

Work material was collected using tools for the study group, including:

- the ICD-10 International Classification of Diseases – diagnosis: F-20 (16);
- the GAF scale - Global Assessment of Functioning scale, a tool for assessing eligibility to participate in the study. The assessment is carried out by a psychiatrist. The scale is commonly used in clinical trials, where the level of functioning is scored on a scale of 0 to 100, with a higher score indicating better functioning. The level of everyday functioning is one of the basic indicators of mental disorder severity. A score above 80 indicates good functioning, while a score between 51 and 80 indicates fairly good functioning (17–19). To determine the recovery criteria, the general functioning on the GAF scale is assumed to be ≥ 50 points (20) and this criterion was chosen as the study qualification criterion;
- the demographic data questionnaire, including: age, gender, education, duration of the disease/relapse episode and the treatment applied. Drug generation: Mixed – treatment with typical first generation and atypical second generation drugs was applied; New – treatment with atypical second generation drugs was applied;
- the Aggression Questionnaire BRAQ by A.H. Buss and M. Perry (1992) is a diagnostic tool commonly used in studies of aggression, assessing aggression in terms of four factors: Physical Aggression (PA), Verbal Aggression (VA), Anger Scale (A) and Hostility Scale (H) (21). The internal consistency coefficient (Cronbach’s alpha) for the questionnaire ranges from 0.68 to 0.94 depending on the gender of the respondents: for men (0.85) and for women (0.77), indicating adequate reliability;
- the State-Trait Anxiety Inventory (STAI), developed by C.D. Spielberger, R.L. Gorsuch, R.E. Lushene (1970) and adapted to the Polish conditions by K. Wrześniewski, T Sosnowski, A. Jaworowska, D. Fecenec (22). This is the most widely used questionnaire for assessing anxiety worldwide. In Poland, it has undergone full adaptation and standardization. The internal consistency coefficient (Cronbach’s alpha) for STAI scores of adult women and men ranges from 0.82 to 0.92 for the state and from 0.76 to 0.90 for the trait, taking into account the age of the study group.

Two scales were used to assess the sense of locus of control, namely Rotter’s I-E scale as the main scale and Drwal’s Delta Scale:

- the questionnaire for measuring the locus of control: J. B. Rotter’s I-E Scale – Locus of control – LOC – the most popular scale used in research on the sense of control, containing 29 pairs of tasks with a forced choice of answers, where the respondent chooses the answer that is more true and closer to their views (3).

The scale is considered satisfactory in terms of estimating reliability and accuracy. However, it has been shown to have certain limitations and, moreover, the influence of social approval, which may lead to a distortion of results, has not been eliminated. Therefore, a second tool was introduced to assess reliability of the scores obtained on Rotter’s I-E Scale – the Delta Questionnaire developed for controlling possible effect of influence of the tendency to distort results by adding to the scale 10 statements from Eysenck’s MPI – Lie Scale (4);

- the standardized Delta Questionnaire (R. Drwal) - Questionnaire on the Level of Sense of Control, containing 24 sentences concerning various traits, preferences and views of the tested individual. It derives from Julian Rotter’s social learning theory (4, 23). The tool with good internal cohesion of questions, with a Cronbach’s alpha coefficient of approx. 0.80, which means that the questionnaire is a reliable tool.

Work material collected using the tools applied for the control group from the healthy population, including

- the demographic data questionnaire, including: age, gender, education;
- the standardized Delta Questionnaire (R. Drwal);
- the questionnaire for measuring the locus of control: J. B. Rotter’s I-E Scale;
- the BPAQ Aggression Questionnaire;
- the STAI scale.

### Data analysis

The analyses were carried out using the IBM SPSS Statistics v. 28.0 software. In the first step, analysis of basic descriptive statistics was carried out. Normality was tested using the Shapiro-Wilk test. Reliability of the questionnaire results was determined using Cronbach’s alpha internal consistency coefficient. Pearson’s r correlation analysis was conducted to determine relationships between the variables. The t-test for independent samples was applied to compare two groups of subjects in terms of the analyzed variables. A. Hayes’ Macro PROCESS v. 4.1 (model 1) was used for moderation analysis. The level of significance was set at α = 0.05.

### Study group characteristics

The study was conducted between 2019 and 2022. Initially, 61 patients diagnosed with schizophrenia according to ICD-10 (sample size calculation: 95% confidence level, maximum error: 12%) who met the criteria and agreed to participate in the research project were included in the study. The study group consisted of patients who were hospitalized in day psychiatric wards and 24-hour rehabilitation wards of the Mazovian Regional Hospital “Drewnica” in Ząbki and the Institute of Psychiatry and Neurology in Warsaw. The inclusion criteria for the study group was age between 18 and 65, no severe psychopathological symptoms of the disease, satisfactory level of cooperation in the treatment process, and an overall functioning assessment of ≥ 50 points in the GAF scale. Thus, the exclusion criterion for the study were severe psychotic symptoms and lack of cooperation in the treatment process.

The healthy control group initially consisted of 61 people from the healthy population, aged 18 to 65 who met the established criteria and agreed to participate in the research project. A non-randomized convenience sample design was used, with the aim of ensuring that the comparison group consisted of individuals who were similar in terms of demographic characteristics to the study group (i.e. age, education, gender). The criterion for inclusion in the study was a signed declaration of no psychiatric or psychotherapeutic treatment. Disclosure during the study of an episode of current or past psychiatric or psychotherapeutic treatment resulted in exclusion from the study. The comparison group consists of healthy individuals who are not receiving treatment for mental disorders, and not individuals with other psychiatric diagnoses, so that the groups can be compared not in clinical, but in social conditions – in search, for members of the therapeutic team working with that group of patients, of the best and most practicable solutions to reduce the risk of aggressive behavior and the possibility of creating targeted rehabilitation programs to improve social functioning, which will lead to longer periods of disease remission.

The paper presents the results of the study group, which consisted of 61 patients: 33 men (54%) and 28 women (46%), and the control group, which consisted of 61 healthy subjects: 20 men (33%) and 41 women (67%). The average age in the study group was 38 (range: 20–65 years of age; SD 11) and in the control group 43 (range: 23–64 years of age; SD 10). In terms of marital status, the study group consisted mainly of bachelors (49%) and single women (26%), while the control group was dominated by married women (44%) and married men (30%). Education: in the study group, 29 individuals (48%) had secondary education and 17 (28%) had a university degree, while 4 patients did not provide this information in the survey. In the control group, the majority had a university degree: 46 individuals (75%), while 11 (18%) had secondary education. The average duration of the disease was 12 ± 9 years. The average number of episodes of disease relapse was 6 ± 4 episodes.

According to the GAF scale, the highest scores were obtained in the group ranging from 61 to 70 points (24 individuals; 20%) and in the group ranging from 71 to 80 points (18 patients; 15%), where the range from 70–61 points describes a patient’s condition in which mild symptoms of the disease, few and transient difficulties in the patient’s functioning appear, but it is a level that allows the patient to function quite well and maintain interpersonal relationships important to them. The range from 80 to 71 is characterized by good functioning, with the possibility of the patient’s response to emerging psychosocial stressors, which are accompanied by slight limitations in functioning, but these are transient responses.

## Results

### Descriptive statistics

**Table 1** presents descriptive statistics of the measured quantitative variables along with the Shapiro-Wilk test, which tests the compliance of the scores with the normal distribution, and the internal consistency coefficient – Cronbach’s alpha, which determines reliability of the results.

**Table 1:** Descriptive statistics with the Shapiro-Wilk test and Cronbach's alpha coefficient.

Variable	*M*	*Me*	*SD*	*Sk.*	*Kurt.*	*Min.*	*Max*	*W*	*P*	Cronbach’s α
Study group										
Physical aggression (PA)	19.84	18.00	6.94	0.87	0.90	9.00	42.00	0.94	0.007	0.733
Verbal aggression (VA)	14.41	14.00	3.83	0.35	0.26	6.00	25.00	0.98	0.242	0.477
Anger (A)	19.00	18.00	6.32	0.28	-0.48	8.00	35.00	0.98	0.409	0.750
Hostility (H)	23.13	24.00	7.11	0.09	-0.45	8.00	39.00	0.99	0.779	0.762
Aggression – overall score	76.38	74.00	18.81	0.50	0.79	37.00	131.00	0.98	0.243	0.869
Anxiety as a state	43.56	41.00	10.42	0.33	-0.64	25.00	67.00	0.97	0.173	0.904
Anxiety as a trait	50.38	50.00	9.63	0.07	-0.56	32.00	71.00	0.98	0.437	0.861
Sense of control	10.90	11.00	4.21	0.35	-0.29	3.00	21.00	0.98	0.252	0.746
EC-LOC	3.43	3.00	2.15	0.20	-0.66	0.00	8.00	0.95	0.017	0.722
IC-LOC	3.25	4.00	1.41	-0.60	-0.44	0.00	5.00	0.90	<0.001	0.523
C-LOC (lies)	5.69	6.00	1.26	0.41	-0.45	4.00	9.00	0.92	<0.001	0.254
Control group										
Physical aggression (PA)	16.72	17.00	4.19	0.25	-0.06	9.00	28.00	0.98	0.496	0.467
Verbal aggression (VA)	14.77	15.00	3.46	0.09	-0.59	9.00	23.00	0.97	0.084	0.549
Anger (A)	17.33	17.00	6.24	0.24	-0.78	7.00	31.00	0.97	0.140	0.807
Hostility (H)	19.97	20.00	6.16	0.05	-0.96	8.00	32.00	0.97	0.126	0.750
Aggression – overall score	68.79	71.00	15.23	-0.26	-0.58	33.00	102.00	0.97	0.231	0.848
Anxiety as a state	35.18	33.00	9.27	0.48	-0.52	21.00	59.00	0.95	0.017	0.926
Anxiety as a trait	39.30	38.00	9.05	0.64	0.44	24.00	68.00	0.97	0.088	0.904
Sense of control	10.64	10.00	4.24	0.37	-0.55	3.00	20.00	0.97	0.082	0.787
EC-LOC	2.13	2.00	2.13	1.09	0.57	0.00	8.00	0.86	<0.001	0.787
IC-LOC	3.49	4.00	1.47	-0.65	-0.79	0.00	5.00	0.86	<0.001	0.648
C-LOC (lies)	5.16	5.00	1.02	0.83	0.47	4.00	8.00	0.86	<0.001	0.157

The analysis showed that most of the analyzed variables were normally distributed in both the study and control groups. A deviation from the normal distribution was observed for the LOC – ZK, WK and K dimensions in both groups, as well as for anxiety as a state in the control group and physical aggression in the study group. Most of the analyzed variables had satisfactory reliability (Cronbach’s α > 0.7).

### Correlations

**Tables 2** and **3** present the correlation matrix of the variables analyzed in the study for the entire sample. The analysis showed that the level of physical aggression was positively correlated with anxiety as a state (weak correlation), as a trait (moderate correlation) and with external locus of control (weak correlation). This means that the higher the level of physical aggression, the higher the level of anxiety as a state and as a trait, and the more external the locus of control. The level of verbal aggression was positively and weakly correlated with the sense of control and weakly and negatively with the lie scale – the higher the level of verbal aggression, the more external the locus of control and the lower the level of social approval. Anger, hostility and general aggression were positively correlated with anxiety as a state and trait, sense of control, EC-LOC and negatively correlated with IC-LOC – with higher levels of anger and hostility, the external locus of control was higher and the internal locus of control was lower (weak or moderate correlations); in addition, the intensity of anxiety as a state and as a trait was also higher. Furthermore, anger and general aggression were negatively and weakly correlated with the lie scale – the higher the level of anger and general aggression, the lower the level of social approval. Anxiety as a state and as a trait was positively correlated with the external locus of control and negatively correlated with the internal locus of control. The sense of control measured with Rotter’s questionnaire was positively and moderately correlated with EC-LOC and negatively correlated with IC-LOC. On the other hand, age, duration of the disease and number of episodes of the disease were positively correlated with EC-LOC (R= 0.316; P=0.027; R=0.311; P= 0.021; R= 0.360; P=0.006, respectively). The number of disease episodes was positively correlated with the sum of STAI X1 (anxiety as a trait) and the sum of STAI X2 (anxiety as a state) (R = 0.287; P= 0.030; R = 0.409; P= 0.002, respectively).

**Table 2:** Correlation matrix of variables included in the standardized scales.

Variable	1	2	3	4	5	6	7	8	9	10	11
1. Physical aggression (PA)	1										
2. Verbal aggression (VA)	0.45**	1									
3. Anger (A)	0.42**	0.55**	1								
4. Hostility (H)	0.33**	0.39**	0.56**	1							
5. Aggression – overall score	0.71**	0.71**	0.84**	0.79**	1						
6. Anxiety as a state	0.29**	0.07	0.36**	0.52**	0.45**	1					
7. Anxiety as a trait	0.32**	0.18	0.50**	0.60**	0.56**	0.75**	1				
8. Sense of control	0.17	0.22*	0.33**	0.48**	0.41**	0.33**	0.41**	1			
9. EC-LOC	0.27**	0.12	0.28**	0.50**	0.42**	0.48**	0.56**	0.47**	1		
10. IC-LOC	-0.16	-0.09	-0.20*	-.26**	-.25**	-0.24**	-0.19*	-0.50**	-0.36**	1	
11. C-LOC (lies)	-0.13	-0.24**	-0.18*	-0.10	-0.20*	0.06	0.01	-0.15	0.01	-0.03	1

*p < 0.05; **p < 0.01.

**Table 3:** Correlation matrix of variables for age, disease duration and number of disease episodes.

Variable	Age		Disease duration		Disease episodes	
	R	P	R	P	R	P
Age	–	–	0.432	0.001	0.059	0.667
Disease duration	0.432	0.001	–	–	0.507	<0.001
Physical aggression (PA)	-0.154	0.248	-0.094	0.482	0.198	0.147
Verbal aggression (VA)	-0.175	0.188	-0.064	0.635	0.098	0.476
Anger (A)	-0.101	0.449	0.022	0.87	0.101	0.463
Hostility (H)	0.065	0.63	-0.002	0.991	0.13	0.344
Aggression – overall score	-0.102	0.447	-0.041	0.76	0.176	0.199
SUM OF STAI X1	0.13	0.333	0.187	0.159	0.287	0.03
SUM OF STAI X2	0.129	0.333	0.224	0.091	0.409	0.002
Rotter’s	0.092	0.491	0.13	0.329	0.111	0.411
EC-LOC	0.316	0.027	0.311	0.021	0.36	0.006
IC-LOC	-0.041	0.759	0.017	0.902	0.014	0.918
C-LOC (lies)	0.07	0.601	-0.176	0.187	-0.11	0.416

### Comparison of patients with schizophrenia and healthy individuals in terms of aggression severity, locus of control and anxiety

In order to compare the results of the two groups – patients and healthy people – t-tests for independent samples were performed. The analysis showed that patients with schizophrenia exhibited higher levels of physical aggression, higher hostility and a higher overall level of aggression than healthy individuals. The strength of the effect for the differences was weak (hostility, general aggression) or moderate (physical aggression).

The subjects in the study group also showed higher levels of anxiety as a state and trait, as well as higher levels of external locus of control and a sense of social approval than healthy subjects. The strength of the effect for the differences was strong for the dimensions of anxiety, moderate for EC-LOC and weak for the lie scale. Results of the analyses are shown in **Table 4**.

**Table 4:** Comparison of groups on the dimensions of aggression, anxiety and locus of control.

Variable	Study group – patients (*n* = 61)		Control group – healthy individuals (*n* = 61)				95% *CI*		
	*M*	*SD*	*M*	*SD*	*t*	*p*	*LL*	*UL*	Cohen’s *d*
Physical aggression (PA)	19.84	6.94	16.72	4.19	3.00	0.003	1.05	5.18	0.54
Verbal aggression (VA)	14.41	3.83	14.77	3.46	-0.55	0.586	-1.67	0.95	0.10
Anger (A)	19.00	6.32	17.33	6.24	1.47	0.144	-0.58	3.92	0.27
Hostility (H)	23.13	7.11	19.97	6.16	2.63	0.010	0.78	5.55	0.48
Aggression – overall score	76.38	18.81	68.79	15.23	2.45	0.016	1.45	13.73	0.44
Anxiety as a state	43.56	10.42	35.18	9.27	4.69	<0.001	4.84	11.91	0.85
Anxiety as a trait	50.38	9.63	39.30	9.05	6.55	<0.001	7.73	14.43	1.19
Sense of control	10.90	4.21	10.64	4.24	0.34	0.732	-1.25	1.78	0.06
EC-LOC	3.43	2.15	2.13	2.13	3.35	0.001	0.53	2.06	0.61
IC-LOC	3.25	1.41	3.49	1.47	-0.94	0.347	-0.76	0.27	0.17
C-LOC (lies)	5.69	1.26	5.16	1.02	2.53	0.013	0.11	0.94	0.46

Moderating role of group membership for the relationship between the dimensions of aggression and sense of control

In order to determine the moderating role of group membership (patients vs. healthy individuals) for the relationship between the dimensions of aggression and sense of control, analyses were performed using A. Hayes’ macro PROCESS v. 4.1 (model 1). The analysis did not confirm the moderating role of group membership for either the sense of control measured by Rotter’s Questionnaire (**Table 5**) or for EC-LOC (**Table 6**) and IC-LOC (**Table 7**). This means that group membership did not differentiate the relationship between the dimensions of aggression and the sense of control.

**Table 5:** Moderating role of group membership for the relationship between the dimensions of aggression and Rotter’s sense of control.

Model/Predictors	X	B	SE	95% CI	R^2^	F
Model 1	Model Physical aggression	0.09	0.07	[-0.06; 0.24]	0.03	1.38
	Group	0.03	0.79	[-1.55; 1.60]		
	Physical aggression x Group	-0.12	0.15	[-0.42; 0.17]		
Model 2	Verbal aggression	0.26*	0.10	[0.05; 0.47]	0.05	2.20
	Group	-0.36	0.75	[-1.84; 1.13]		
	Verbal aggression x Group	-0.07	0.21	[-0.48; 0.34]		
Model 3	Anger	0.22***	0.06	[0.11; 0.34]	0.11	4.96**
	Group	0.11	0.73	[-1.34; 1.56]		
	Anger x Group	-0.06	0.12	[-0.29; 0.17]		
Model 4	Hostility	0.30***	0.05	[0.20; 0.41]	0.23	11.98***
	Group	0.70	0.70	[-0.68; 2.08]		
	Hostility x Group	-0.03	0.10	[-0.23; 0.18]		
Model 5	Overall aggression	0.10***	0.02	[0.06; 0.14]	0.17	8.13***
	Group	0.50	0.72	[-0.93; 1.93]		
	Overall aggression x Group	-0.01	0.04	[-0.10; 0.07]		

*p < 0.05; **p < 0.01; ***p < 0.001.

**Table 6:** Moderating role of group membership for the relationship between the dimensions of aggression and EC-LOC.

Model/Predictors	X	B	SE	95% CI	R^2^	F
Model 1	Physical aggression	0.07	0.04	[-0.01; 0.15]	0.13	5.76**
	Group	-1.07**	0.40	[-1.86; -0.27]		
	Physical aggression x Group	-0.02	0.08	[-0.17; 0.12]		
Model 2	Verbal aggression	0.08	0.05	[-0.03; 0.19]	0.11	4.75**
	Group	-1.32**	0.39	[-2.09; -0.56]		
	Verbal aggression x Group	-0.07	0.11	[-0.29; 0.14]		
Model 3	Anger	0.09**	0.03	[0.03; 0.15]	0.15	6.95***
	Group	-1.15**	0.38	[-1.90; -0.40]		
	Anger x Group	-0.05	0.06	[-0.17; 0.07]		
Model 4	Hostility	0.15***	0.03	[0.10; 0.20]	0.29	15.80***
	Group	-0.82*	0.35	[-1.52; -0.11]		
	Hostility x Group	0.01	0.05	[-0.10; 0.11]		
Model 5	Overall aggression	0.05***	0.01	[0.02; 0.07]	0.22	10.91***
	Group	-0.94*	0.37	[-1.68; -0.21]		
	Overall aggression x Group	-0.01	0.02	[-0.05; 0.03]		

*p < 0.05; **p < 0.01; ***p < 0.001.

**Table 7:** Moderating role of group membership for the relationship between the dimensions of aggression and IC-LOC.

Model/Predictors	X	B	SE	95% CI	R^2^	F
Model 1	Physical aggression	-0.04	0.03	[-0.09; 0.01]	0.03	1.17
	Group	0.13	0.27	[-0.41; 0.67]		
	Physical aggression x Group	0.01	0.05	[-0.10; 0.10]		
Model 2	Verbal aggression	-0.03	0.04	[-0.11; 0.04]	0.02	0.69
	Group	0.26	0.26	[-0.26; 0.78]		
	Verbal aggression x Group	0.03	0.07	[-0.11; 0.17]		
Model 3	Anger	-0.04*	0.02	[-0.08; -0.01]	0.05	1.95
	Group	0.17	0.26	[-0.34; 0.69]		
	Anger x Group	-0.03	0.04	[-0.11; 0.05]		
Model 4	Hostility	-0.06**	0.02	[-0.10; -0.02]	0.07	3.12*
	Group	0.07	0.26	[-0.45; 0.58]		
	Hostility x Group	-0.02	0.04	[-0.10; 0.05]		
Model 5	Overall aggression	-0.02**	0.01	[-0.04; -0.01]	0.06	2.73*
	Group	0.09	0.26	[-0.43; 0.61]		
	Overall aggression x Group	-0.01	0.01	[-0.04; 0.02]		

*p < 0.05; **p < 0.01; ***p < 0.001.

### Moderating role of group membership for the relationship between the dimensions of anxiety and sense of control

In order to determine the moderating role of group membership for the relationship between anxiety and sense of control, an analysis was performed using A. Hayes’ macro PROCESS v. 4.1 (model 1). The analysis confirmed a significant moderating role of group membership for the relationship between anxiety as a state and internal locus of control (IC-LOC) (**Table 8**). In patients with schizophrenia, the relationship between internal locus of control and anxiety as a state proved insignificant (b = 0.003; p = 0.879; 95% CI [-0.03; 0.04]), whereas in the group of healthy individuals, this relationship was negative (b = -0.08; p < 0.001; 95% CI [-0.12; -0.04] – with a higher level of anxiety as a state, the level of internal locus of control was lower.

**Table 8:** Moderating role of group membership for the relationship between the dimensions of anxiety and sense of control.

Y	X	B	SE	95% CI	R^2^	F
Sense of control (Rotter)	Anxiety as a state	0.15***	0.04	[0.08; 0.23]	0.13	5.65**
	Group	1.01	0.79	[-0.54; 2.57]		
	Anxiety as a state x Group	0.03	0.07	[-0.12;0.17]		
Sense of control (Rotter)	Anxiety as a trait	0.21***	0.04	[0.14; 0.28]	0.21	10.69***
	Group	2.05*	0.80	[0.47; 3.64]		
	Anxiety as a trait	0.02	0.07	[-0.13; 0.17]		
EC-LOC	Anxiety as a state	0.09***	0.02	[0.06; 0.13]	0.25	12.97***
	Group	-0.53	0.38	[-1.29; 0.24]		
	Anxiety as a state x Group	0.04	0.04	[-0.04; 0.11]		
EC-LOC	Anxiety as a trait	0.11***	0.02	[0.08; 0.15]	0.31	17.66***
	Group	-0.04	0.39	[-0.82; 0.74]		
	Anxiety as a trait	0.01	0.04	[-0.06; 0.08]		
IC-LOC	Anxiety as a state	-0.04**	0.01	[-0.06; -0.01]	0.14	6.20**
	Group	-0.07	0.27	[-0.60; 0.45]		
	Anxiety as a state x Group	-0.08**	0.02	[-0.13; -0.03]		
IC-LOC	Anxiety as a trait	-0.03*	0.01	[-0.05; -0.01]	0.06	2.44
	Group	-0.06	0.30	[-0.65; 0.53]		
	Anxiety as a trait	-0.04	0.03	[-0.10; 0.01]		

*p < 0.05; **p < 0.01; ***p < 0.001.

Statistically significant differences were found between the number of relapses and disease duration (P=0.001); level of physical aggression (P=0.043); level of anxiety as a trait (P=0.026) and as a state (P=0.008); and external locus of control (0.012). Clinically significant differences were also shown in terms of hostility severity (P=0.260), where the level of hostility increased with the number of disease episodes.

**Table 9** shows a comparative analysis of the GAF scale scores with clinical data. Statistically significant differences were shown between the GAF scale and the level of anxiety as a trait and hostility severity (P=0.048), but without specific clinical significance.

**Table 9:** Comparative analysis of GAF scale scores with clinical data.

GAF	60-51	70-61	80-71	90-81	All	P
Age	41.2 ± 10.9	37.5 ± 9.3	33.8 ± 11.8	43.6 ± 16.3	37.8 ± 11.2	0.176
Disease duration	14.0 ± 8.2	10.3 ± 7.1	11.4 ± 11.4	14.3 ± 8.8	11.8 ± 8.9	0.619
Physical aggression (PA)	20.2 ± 6.0	21.1 ± 7.8	18.7 ± 6.6	16.8 ± 6.6	19.8 ± 6.9	0.516
Verbal aggression (VA)	14.6 ± 3.5	15.7 ± 4.2	13.4 ± 3.3	11.4 ± 2.6	14.4 ± 3.8	0.063
Anger (A)	18.1 ± 5.8	20.6 ± 6.8	17.7 ± 6.7	18.6 ± 1.7	19.0 ± 6.3	0.469
Hostility (H)	23.9 ± 6.8	25.6 ± 7.4	19.6 ± 6.5	22.0 ± 3.8	23.1 ± 7.1	0.048
Aggression – overall score	76.9 ± 12.3	83.0 ± 20.7	69.3 ± 19.7	68.8 ± 11.8	76.4 ± 18.8	0.092
SUM OF STAI X1	49.9 ± 11.0	42.7 ± 10.2	39.7 ± 9.1	43.8 ± 8.4	43.6 ± 10.4	0.048
SUM OF STAI X2	52.6 ± 8.9	52.2 ± 10.7	47.3 ± 9.0	46.4 ± 5.9	50.4 ± 9.6	0.239
Rotter’s SUM	11.1 ± 4.5	11.2 ± 4.5	10.1 ± 3.8	12.0 ± 4.3	10.9 ± 4.2	0.758
EC-LOC	3.6 ± 1.8	3.9 ± 2.3	2.5 ± 2.2	4.0 ± 1.2	3.4 ± 2.1	0.180
IC-LOC	3.4 ± 1.4	3.1 ± 1.5	3.3 ± 1.4	3.4 ± 1.1	3.2 ± 1.4	0.916
C-LOC (lies)	5.9 ± 1.4	5.5 ± 1.3	5.7 ± 1.2	5.6 ± 1.1	5.7 ± 1.3	0.839
Drug generation						
New, n (%)	8 (73)	20 (91)	14 (82)	4 (80)	46 (84)	
Mixed, n (%)	3 (27)	2 (9)	3 (18)	1 (20)	9 (16)	0.599

The scores are presented as average ± standard deviation.

## Discussion

According to the statistical analyses, the group of patients diagnosed with schizophrenia showed a statistically significant higher level of aggression compared with the control group of healthy individuals. The statistically significant higher level of aggression in the patient group concerned active aggression as well as the score of hostility and the overall level of aggression. The results confirmed that patients with schizophrenia tend to present a more external locus of control, compared with healthy control group, and this was associated with higher levels of aggression.

Given the innovative nature of this study, it will be difficult to relate to the results of other studies conducted in Poland or worldwide, consistent in terms of their subject matter, which would involve a comparative analysis of the occurrence of aggression in correlation with the sense of locus of control in patients diagnosed with schizophrenia in relation to a control group from the healthy population, where the test group would consist of patients in remission of psychopathological symptoms, hospitalized in day and therapeutic psychiatric wards. One can only sequentially refer to the scores obtained in relation to individual variables such as aggression, sense of locus of control or the occurrence of anxiety in schizophrenia, as well as their interdependence.

Most of the studies conducted so far focus on the analysis of aggression in relation to the disease and/or the sense of locus of control depending on the disease, the impact of the sense of locus of control on the study and the occurrence of aggressive behaviors in the group of adolescents or perpetrators of aggressive offences. Many researchers devote their attention to issues indicating the occurrence of dependencies between a higher level of aggressiveness depending on the disease phase and the occurring psychopathological symptoms or anxiety severity among patients diagnosed with schizophrenia. There are also reports suggesting that the locus of control ought to be considered in relation to the diagnosis of psychiatric disorders, the patient’s functioning and recovery, as well as their personality.

The results of the author’s own research partially coincide with the results of the study by Harrow, Handsford and Astrachan-Flecher (13). The study was based on data from the Chicago Follow-Study. In the cited study, results were obtained in the assessment of the locus of control, where patients diagnosed with schizophrenia, after going through the acute phase of the disease and severity of psychopathological symptoms, did not present a more external locus of control compared with other study groups. On the other hand, an internal locus of control was associated with an increased recovery rate in schizophrenia (p<0.05). A statistically significant external locus of control was associated with depression (p=0.01), and an association between the external control and psychosis was also observed (p<0.05). The results of this analysis demonstrate that patients with schizophrenia who have a more internal locus of control have a greater chance of recovery. The hypotheses that recovery was more frequently achieved by patients with schizophrenia who presented a more internal locus of control, had consecutive periods of recovery within the 15 years (χ 2 = 4.58, 1 df, p <0.05), than patients with schizophrenia with a sense of external locus of control proved justified. The results were similar for the whole sample of patients (χ 2 = 6.50, 1 df, p = 0.01). The author’s own findings are consistent with the result obtained by Harrow, Handsford and Astrachan-Flecher regarding the statistical significance of control, anxiety and hostility. As the study showed, notably the patients who had an external locus of control were significantly more likely to be patients with high levels of anxiety as a trait (r = 0.25, 125 df, p < 0.01), to be hostile (r = 0.19, 125 df, p < 0.05), to show higher disorganization (r = 0.44, 122 df, p < 0.001) and to have lower self-esteem (r = 0.29, 123 df, p < 0.01).

Another study that explored aggression in patients with schizophrenia and the accompanying experiences of the patient showed a link between psychotic experiences and the severity of hostility and aggressiveness, where accompanying anxiety was important for the patient’s feelings, as well as the feeling of an unreal external world and of being misunderstood and alienated in it. The study by Konstantinos Trisigotis and Wojciech Gruszczyński (2013) (24) focused on investigating the relationship between psychotic symptoms and aggressiveness in patients suffering from schizophrenia. It showed a statistically significant correlation between variables such as schizophrenia and hostility: and aggression. Based on the results of the study, a close relationship can be observed between aggressive behaviors and psychotic experiences, i.e.: hallucinations, delusions, strange sensory experiences, loss of ego control in the sphere of control, loss of ego control in the cognitive sphere, with accompanying sensitivity and suspiciousness as a result of those psychotic experiences. Research confirms that they cause the patient to be constantly vigilant and experience a distorted sense of security accompanied by anxiety. In an attempt to adapt, the patient avoids the threatening world with a hostile attitude towards the outside world. Recent work published by Bravve et al. (2025) underscores that aggressive behavior in schizophrenia may mask latent suicidal ideation, and that elevated aggression and trait anxiety serve as markers of emotional dysregulation. Their findings support the notion that internalized aggression is a clinically relevant marker, particularly in patients with high trait anxiety (25).

Another scientific report by the same research group (26) provides an answer to the question regarding the relationship between the use of new generation neuroleptics (risperidone) and the use of classic neuroleptics in a group of schizophrenia patients, and subjectively perceived, clinically latent aggression and hostility. The study group consisted of 60 patients diagnosed with paranoid schizophrenia (the most common form), with a documented 5-year duration and treatment of schizophrenia, both inpatient and outpatient. One half of the examined group of patients were patients without severe psychotic symptoms, aged 25-65, treated with the latest generation neuroleptic Risperidone, while the other half were patients with schizophrenia, aged 26-65, treated with a classic antipsychotic drug. The MMPI (Minnesota Multiphasic Personality Inventory) questionnaire and its selected scales and subscales were used as the diagnostic and psychological tool. The study results obtained indicated statistically significant differences in the perceived hostility and aggressiveness in patients diagnosed with schizophrenia treated with a classical neuroleptic and risperidone – an atypical antipsychotic. Furthermore, the study showed that patients treated with risperidone also scored lower in terms of perceived aggressiveness and hostility than the group of patients treated with a classic neuroleptic. In addition, reduced intensity of psychopathological symptoms was observed in the group of patients treated with a new generation neuroleptic. This pharmacological differentiation aligns with neuroimaging research from the ENIGMA consortium (2023), which demonstrated structural differences in prefrontal and frontotemporal regions associated with higher aggression scores and external LOC in patients with schizophrenia. The authors concluded that deficits in cognitive control and emotion regulation circuits may partially mediate the LOC-aggression relationship (27).

In another clinical study, Harrow, M. and Ferrante, A. (1969) (7) used Rotter’s I-E scale (LOC-control) to examine 128 patients in psychiatric hospitals with various psychiatric diagnoses who were in an acute state of the disease, i.e. with severe psychopathological symptoms, who were examined in the 1st and then in the 7th week of hospitalization. The results obtained showed that patients with schizophrenia placed control more externally than patients with other psychiatric diagnoses (p < 0.02). Older patients with schizophrenia placed a sense of control more internally than younger patients (p < 0.05). The results of the Rotter’s I-E (LOC-control) scale correlated significantly with the scores concerning self-confidence and frustration. The results in the acute group suggested that people with more severe psychopathology and weaker social skills (schizophrenia, younger patients and, to a small extent, men) had a more external locus of control. In the 7th week of hospitalization, the scores on Rotter’s I-E scale for schizophrenia did not change significantly. However, people with depression became more internal (p < 0.02). Patients with schizophrenia differed from the group of patients with other psychiatric diagnoses in terms of change score (p < 0.001), and the group of female subjects showed more internal locus of control (p < 0.01).

The scientific report by Hanna Levenson of the University Hospital of Texas (28) analyzed the relationship between the sense of locus of control in relation to the groups of subjects: psychotic patients (schizophrenia as a non-differentiated type and paranoia, depression and neuroticism) and a group of healthy subjects. Levenson examined 165 patients of a psychiatric hospital in terms of their functioning and their sense of locus of control (Rotter’s I-E). The study showed that hospitalized patients diagnosed with schizophrenia scored significantly higher and presented a significantly more external sense of control, and were more likely to believe in the control of their lives by other powerful and unknown forces of fate than neurotic individuals (p < 0.01, p < 0.05). Interestingly, patients readmitted to the hospital had higher scores for perceived control by chance external forces on their next hospitalization than new patients on their first hospitalization (p < 0.05). In light of these findings, Tusconi and Dursun (2025), in an editorial published in Frontiers in Psychiatry, emphasize the importance of combining cognitive-behavioral strategies with structured social rehabilitation programs in reducing aggression and strengthening internal control beliefs in patients with psychotic disorders (29).

Considering the scientific reports presented above and the results obtained in the author’s own study, it seems reasonable to conclude that patients treated for schizophrenia show significantly higher levels of aggressive behaviors, such as physical aggression and hostility, compared with healthy individuals. The subjects also obtained statistically significant higher scores in terms of accompanying level of anxiety as a state and a personality trait, which may suggest that they perceive threatening factors more quickly and more extensively than healthy individuals. Noteworthy is the observation, confirmed in many studies, that patients diagnosed with schizophrenia place their locus of control much more externally than healthy people or people with other mental disorders, and this variable is still statistically significant among patients, even after achieving a state of remission of psychopathological symptoms. The research I have conducted confirms this relationship. These results suggest that the subjects, after completing therapeutic and rehabilitation treatment, may present significantly more favorable results in terms of locus of control, accompanying anxiety, and may be characterized by less suspiciousness, distrust, hostility and a greater belief in their own abilities than during the rehabilitation process. Therefore, the issue is worth further exploration. However, the way in which the two groups – the study group and the control group – perceive themselves seems to be important. The statistically significant higher score in the C-LOC (lies) variable obtained in the study group may indicate an aspiration, a tendency to present oneself in a more favorable light. Therefore, one can conclude that patients seek social approval, which may positively influence their engagement in treatment. Consent to further treatment in a day or rehabilitation ward may suggest that the patient realizes the benefits of the stay and the need to learn to adapt to social expectations.

The study carried out so far does not fully exhaust the subject matter being analyzed. It would be worthwhile to continue the research based on a wider spectrum of variables, by including, for example, other diagnostic groups in the analysis in order to check significance of the correlations in the variables taken up in the study and whether or not the results presented herein will be confirmed. Patients suffering from a mental disease perceive the disease itself as a threat, perhaps even greater than any other traumatic experiences that make them feel unsafe. This may influence the locus of control and, consequently, the patient’s recovery process.

The results obtained suggest that it is worth looking for opportunities and ways of improving patients’ own effectiveness in functioning and coping with everyday difficulties so that it is not chance or fate that influences the lives of those patients, but the mechanisms of coping with anxiety and illness learned in therapeutic training and rehabilitation which would allow patients to achieve better functioning and long disease remissions. The inclusion of a team of professionals: nurses, doctors, psychologists and therapists whom the patient trusts, promises better cooperation in the rehabilitation process of this group of patients and more favorable treatment outcomes.

The available literature on the subject suggests that maintaining continuity of treatment and early inclusion of the patient in a comprehensive rehabilitation program offers the chance to strengthen and develop the patient’s resources, increases the chance of maintaining remission, improving social functioning and striving to achieve well-being in this group of patients.

### Limitation

After statistical analyses (chi2 test) of the demographic characteristics of the study group and the control group, statistically significant differences were found in terms of: gender (P=0.017); education (P <0.001) and age (P =0.006). However, the above demographic characteristics did not have a statistically significant impact on the level of anxiety and aggression, either in the study group or in the healthy group (for all characteristics: R <0.1; P>0.05).

The study carried out is cross-sectional in nature, which can have potential drawbacks and is subject to greater error. A longitudinal study with an analytical perspective could have been less prone to error and would have allowed for the establishment of cause-and-effect relationships precisely because of the time perspective taken. However, the epidemiological situation encountered during the study, related to SARS-CoV-2, was an additional obstacle preventing extension of the study to include patients with schizophrenia. It was a challenge for the researcher due to the inability to reach the patient and closure of rehabilitation and day care wards. The post-Covid state, on the other hand, required a special approach to the patient in order to obtain their consent for the examination. Anxiety and a sense of danger, distrust of strangers that is at the heart of the disease itself, have been intensified by the experience of imposed social isolation and related restrictions. In the future, under more favorable circumstances, I would like to continue and explore the topic, in order to expand the study.

Cronbach’s alpha for some tests was <0.6. This fact limits the study. However, it is due to the application of tests of varying difficulty in a heterogeneous group of patients. In this situation, it is often difficult to obtain an alpha value > 0.6.

## Conclusions

1. The results obtained confirmed the research hypothesis: a higher level of aggression occurs in patients who place their locus of control externally.
2. Analysis results showed a significant relationship between the external locus of control and all dimensions of aggression: physical aggression (correlation with EC-LOC), verbal aggression (correlation with Rotter’s sense of control), anger (correlation with EC-LOC and IC-LOC), hostility (correlation with EC-LOC and IC-LOC) and overall level of aggression (correlation with EC-LOC and IC-LOC).
3. Statistically significant correlations were confirmed between higher levels of anxiety as a state and as a trait in patients compared with healthy individuals.
4. In the study group, the higher the level of physical aggression, the higher the level of anxiety as a state and as a trait, and the more external the locus of control.
5. Furthermore, the analysis showed that in the study group, the higher the level of verbal aggression, the more external the locus of control and the lower the level of social approval.
6. In the study group, the higher the level of anger and overall aggression, the lower the level of social approval.
7. The analysis revealed that with a higher level of anger and hostility in the group of schizophrenic patients, the external locus of control was higher, the internal locus of control was lower and the severity of anxiety as a state and as a trait was also higher.
8. No statistically significant differences were found between the GAF scale and the clinical data, and the patient assessment scales used.
9. The analyses showed that the patients’ higher education level had a protective effect on the severity of physical aggression and the overall aggression score.
10. The results of the study confirmed that the number of relapses was related to the level of physical aggression, the level of anxiety as a trait and state, and the external locus of control.
11. Monitoring the severity of aggressive behaviors in the course of the disease could enable the creation of better adapted therapeutic programs.

## Data Availability

The raw data supporting the conclusions of this article will be made available by the authors, without undue reservation.
